# Recent Researches of Prof. Agassiz

**Published:** 1853-08

**Authors:** 


*Recent Researches of Prof. Agassiz.*—Prof. Agassiz has recently made a rapid tour from Charleston, S. Carolina, through Alabama, Mississippi, and Louisiana, thence up the Mississippi to St. Louis, Chicago, and along by the Great Lakes to New York and Massachsetts. In a recent letter from him, addessed to J. D. Dana, dated Cambridge, June 9th, he mentions the following as some of the results of his tour.

"'I have been successful in collecting specimens, especially fishes, of which I have brought home not less than *sixty* new species, mostly from the great southern and western rivers. Some of these are particularly interesting. I would mention foremost a new genus, which I shall call *Chologaster*, very similar in genearl appearance to the blind fish of the Mammoth Cave, though provided with eyes; it has like Amblyopsis *the anal aperture* far advanced *under the throat*, but is *enirely deprived of veutral fins*; a verystrange and unexpected combination of characters. I know but one species, Ch. cornutus, *Ag*. It is a small fish scarely three inches long, living in ditches of the rice fields in South Carolina. I derive its specific name from the singular form of the snout, which has two hornlike projections above.

The family of Cyprinodonts has received the most numerous additions, and among them there are again new combinations of charcters. Several years ago I noticed two species of a new genus which I would call *Heterandria*, from the great difference observed between the two sexes, the males having the venetral fins near the pectorals in about the same position as in the *Thoracic* fishes, while the females have those fins in the middle of the belly as in the *Abdominals*. Of this genus I have observved several new species They all live in dense schools in shallow waters. Another type, *Zygonectes*, presents no such sexual differences, and differs also in its habits. These fishes are constantly seen swimming on the top of water *in pairs*, whence their name. I have found half a dozen new species of this genus.

You may remember the remarkble genus Mollinesia described by Lesueur from specimens obtained from Lake Ponchartrain and from Florida. If you do not, pray look for the figures in the Journal of the Acad. of Nat, Sci., vol. 2, to appreciate the facts here mentioned. From its structure and from the sexual differences observed among other Cyprinodonts, I have long entertained the opinion that this genus had been established upon the males of Pœcilla mutilineata also described by Lesueur (same Journal), and both admitted as distinct in the great natural history of fishes by Cuvier and Valenciennes. Having found both together in all the Gulf States, I have watched them carefully, and in Mobile as well as in New Orleans, I have seen them day after day in copulation during the months of April and May; so that their specific identity is now an established fact. I have caught hundreds of them and found all the Pœcilias to be females and all the Mollinesias males; and what is further very interesting, the females are viviparous. I have been able to trace their whole embryonic development in the body of the mother, in selecting specimens in different stages of gestation.

I do not remember whether I have already mentioned to you the existence in the United States of two families of fishes not before observed in our waters, one the *Myxinoids* with one species from Eastport in Maine, collected by W. Stimpson, the other the *Erythrinoids* of Valenciennes, or *Charaxini* without adipose fin of T. Müller of which a new genus occurs in the freshwaters of our northern and middle as well as western States, with half a dozen species, some of which have been unforunately described as Lucicus, Fundulus and Hydrargyra, with which genera they have no affinity, while other new ones have been discovered by Professor Baird and myself. I shall call this genus *Melanura*, from the singular black mark which all species show upon the tail. But I would tire you were I to go on with my ichthyological remarks, even if I should limit myself to enumerating new genera, for I have many more of these.

I will close this long letter with one observation upon Crustacea, which may have a more immediate interest for you, if you have not yet noticed the fact yourself. On my return from Florida two years ago I noticed among many specimens of Lupa dicantha, one in which the tail presented a triangular form intermediate between that of the male and that of the female. Unable to ascertain from a single specimen whether it was a mere variety or perhaps an improperly developed female, I awaited another opportunity for a fuller investigation, which the market of Charleston, S. C. afforded largely during the latter part of February last, when I ascertained that that form was at times as common in the market as either the males or the females, and upon careful anatomical examination, I satisfied myself farther, that these specimens *are entirely deprived of internal sexual organs*, though slight indications of the openings of the sexual or gans, entirely closed up by the calcareous matter, clearly indicated that they are imperfectly developed females, a kind of neuters among crabs, the great number of which leads to the supposition that they are not without function in the general economy of these animals. The tail is soldered to the carapace, the last joints only at which the alimentary canal terminates, being moveable. At the same time males and females were dissected, and showed the sexual organs in that fullness which precedes copulation. Looking afterwards for similar conditions in other species, I found in the collection of Professor L. Gibbes, specimens of L[illegible]pa cribraria, and of L. Sayi with the same conformation of their tail.

I cannot help returning to my fishes to say, that I have now *twenty species* of Lepidosteus from the United States in my collection, a good foundation upon which to base a revision of the fossil fishes. I could not say how many other new things I have collected, for I have not yet unpacked half my packages."—*Silliman's Jour.*

